# Pancreatic Beta Cells Synthesize Neuropeptide Y and Can Rapidly Release Peptide Co-Transmitters

**DOI:** 10.1371/journal.pone.0019478

**Published:** 2011-04-29

**Authors:** Matthew D. Whim

**Affiliations:** Department of Cell Biology and Anatomy, Louisiana State University (LSU) Health Sciences Center, New Orleans, Louisiana, United States of America; University of Bremen, Germany

## Abstract

**Background:**

In addition to polypeptide hormones, pancreatic endocrine cells synthesize a variety of bioactive molecules including classical transmitters and neuropeptides. While these co-transmitters are thought to play a role in regulating hormone release little is known about how their secretion is regulated. Here I investigate the synthesis and release of neuropeptide Y from pancreatic beta cells.

**Methodology/Principal Findings:**

NPY appears to be an authentic co-transmitter in neonatal, but not adult, beta cells because (1) early in mouse post-natal development, many beta cells are NPY-immunoreactive whereas no staining is observed in beta cells from NPY knockout mice; (2) GFP-expressing islet cells from an NPY(GFP) transgenic mouse are insulin-ir; (3) single cell RT-PCR experiments confirm that the NPY(GFP) cells contain insulin mRNA, a marker of beta cells. The NPY-immunoreactivity previously reported in alpha and delta cells is therefore likely to be due to the presence of NPY-related peptides. INS-1 cells, a beta cell line, are also NPY-ir and contain NPY mRNA. Using the FMRFamide tagging technique, NPY secretion was monitored from INS-1 beta cells with high temporal resolution. Peptide release was evoked by brief depolarizations and was potentiated by activators of adenylate cyclase and protein kinase A. Following a transient depolarization, NPY-containing dense core granules fused with the cell membrane and discharged their contents within a few milliseconds.

**Conclusions:**

These results indicate that after birth, NPY expression in pancreatic islets is restricted to neonatal beta cells. The presence of NPY suggests that peptide co-transmitters could mediate rapid paracrine or autocrine signaling within the endocrine pancreas. The FMRFamide tagging technique may be useful in studying the release of other putative islet co-transmitters in real time.

## Introduction

Located within the islets of Langerhans, pancreatic beta cells synthesize the hormone insulin. A rise in the level of blood glucose triggers beta cell activity and an increase in insulin secretion. Consequently glucose uptake by insulin-sensitive cells is accelerated and the circulating levels of glucose are homeostatically adjusted. However in addition to insulin, beta cells also synthesize a range of other bioactive molecules. These include small molecule classical transmitters such as ATP and GABA and larger molecules including peptides [1], [2], [3], [4]. It is thought that such transmitters play a role in modulating the activity of islet cells, including beta cells [5]. For example GABA is secreted from beta cells and activation of islet GABA_B_ receptors can regulate the secretion of insulin [6]. However while the existence of beta cell co-transmitters has been recognized for some time, the factors that govern their release are not entirely clear.

Neuropeptide Y (NPY) is an example of a pancreatic co-transmitter that seems to have a modulatory role. NPY is found in pancreatic islets [7], [8] and its exogenous application suppresses insulin release from perfused islets [9]. In NPY knockout mice the circulating levels of insulin become abnormally elevated when the animals are fed *ad lib* [10]. Finally, deletion of the NPY1 receptor leads to an increase in plasma insulin levels [11]. These results are consistent with the prevailing idea that NPY is an autocrine or paracrine inhibitory co-transmitter within the pancreas.

However a number of issues remain unclear. First, which islet cells contain NPY? Some studies have concluded that NPY is primarily expressed in beta cells [4], [12], [13] while others have reported that it is found in the glucagon secreting alpha cells [14] or in the somatostatin-containing delta cells [15]. Because the NPY-related peptides, pancreatic polypeptide and PYY are also present in islets it has been suggested that some of the reported NPY-ir could be due to the presence of these other peptides [4]. NPY-ir is also found in neuronal processes within the pancreas [16]. Second, how is the release of these co-transmitters regulated? Peptides like NPY and proteins like insulin are synthesized *via* the endoplasmic reticulum and packaged into dense core granules as they move through the *trans*-Golgi. Previous studies have shown that when insulin-containing dense core granules fuse with the cell membrane, the release of insulin can be delayed for hundreds of milliseconds, which may be related to a slow dissolution of the insulin-containing dense core [17]. Conversely small molecules like ATP and GABA are released unhindered [18]. Whether the secretion of neuropeptides like NPY show a delayed release is not known.

To address these questions I have used a variety of approaches to confirm that NPY expression is confined to mouse beta cells during early post-natal development. By examining the distribution of NPY immunoreactive cells in wild type, NPY knockout and NPY(GFP) animals I conclude that this peptide is not expressed in alpha, delta or F cells. Because the INS-1 beta cell line also synthesizes NPY, the kinetics of NPY release was then monitored from these cells using the FMRFamide tagging technique. The advantage of this electrophysiological approach is that it can measure secretion with millisecond resolution. I find that most peptidergic secretory events are brief suggesting that peptides like NPY can be rapidly liberated from fusing dense core granules. Application of the FMRFamide tagging technique may be useful in future studies examining the release of similar co-transmitters from other pancreatic endocrine cells.

## Results

### Neuropeptide Y is expressed in mouse pancreatic beta cells

**Figure 1:**
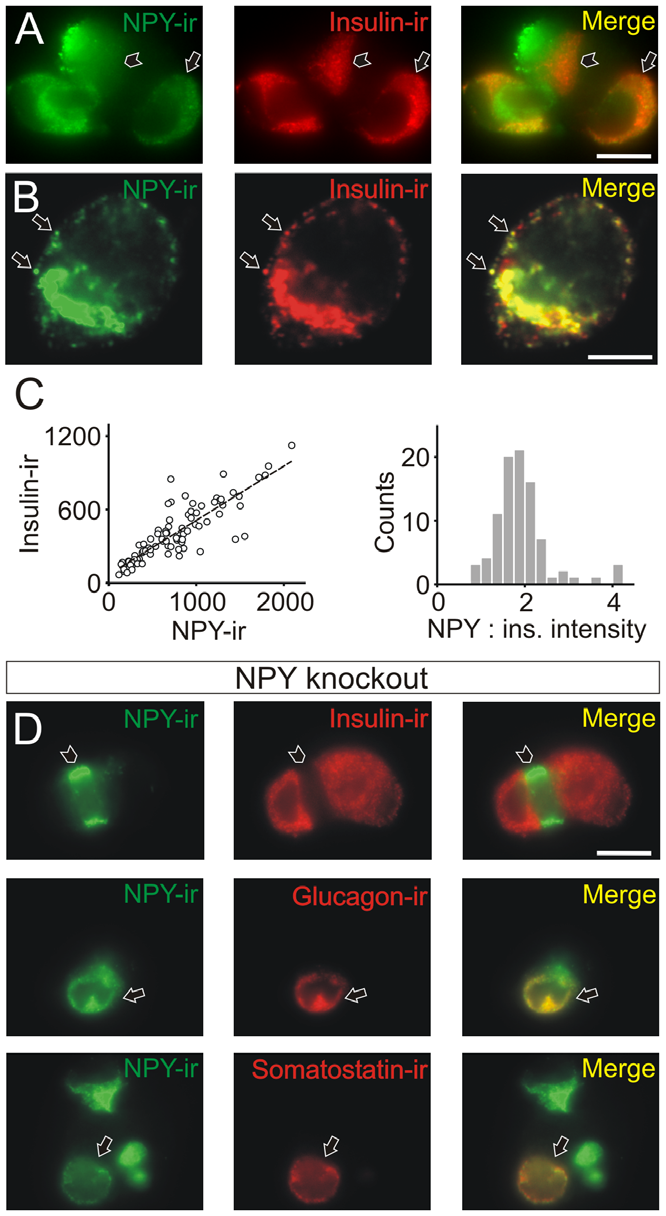
Neuropeptide Y is synthesized by mouse pancreatic beta cells. (A). A small cluster of pancreatic cells in which some insulin-ir (beta) cells are also NPY-ir (arrow indicates one example). Also present is a beta cell that is not NPY-ir (arrow head). (B). Single confocal slice through an insulin-ir cell that was also NPY-ir. Indicated are two puncta that are co-stained for NPY and insulin (arrows). (C). Quantification of the fluorescent signal from individual puncta. ROI's corresponding to single NPY-ir puncta were selected then the corresponding signal in the insulin (red) channel was also measured. Left panel; a plot of fluorescence intensities indicates that there is a positive correlation between the signal in the two channels consistent with the coexpression of NPY and insulin in individual granules (n = 90 granules from 3 cells, R = 0.86). Right panel; the distribution of the punctal signals. (D). Islet cells from a neonatal (P0) NPY knockout animal were co-stained for NPY, insulin, glucagon and somatostatin. NPY-ir was not present in insulin-ir cells (arrow head), while punctate “NPY-ir” was seen in both glucagon- and somatostatin-ir cells (arrows). Thus authentic NPY-ir is only present in beta cells. Scale bars 10 µm (A,D) 5 µm (B).

When dissociated cultures of pancreatic cells from neonatal mice (P0–P2) were co-stained for NPY and insulin, a population of cells was identified that contained punctate NPY-immunoreactivity (NPY-ir) as expected for a transmitter located in the regulated secretory pathway (Figure 1A). Most insulin-ir cells were also NPY-ir (70%; 19/27 cells). To determine if NPY and insulin were present in the same granules, the red and green fluorescent signals from single puncta were quantified. Many puncta were co-labeled (Figure 1B) and there was a positive correlation (R = 0.86) in the intensity of the NPY- and insulin-ir (Figure 1C, left panel). A histogram of the intensity ratios also had a dominant peak suggesting that the majority of NPY-containing granules contained insulin (Figure 1C, right panel).

As seen in Figure 1A, some of the NPY-ir cells were not insulin-ir and therefore to determine which islet cells expressed NPY the cultures were co-stained for insulin, glucagon and somatostatin (possible co-existence of pancreatic polypeptide and NPY was tested using a transgenic approach; see below). In neonatal animals NPY-ir was co-localized with insulin-, glucagon- and somatostatin-immunoreactivity (not shown). When islet cells from adult mice (P25) were examined, a somewhat different picture emerged. In these animals NPY-ir was still found in glucagon- and somatostatin-ir cells, but *not* in insulin-ir cells (not shown). These results suggested that (1). NPY expression was developmentally regulated or (2). the NPY antibody was also recognizing the related peptides, PYY and PP, which are known to be present in pancreatic islets [4]. To test the latter possibility, NPY-ir was examined in neonatal islet cells isolated from an NPY knockout mouse. In these cultures, although no NPY-ir was observed in insulin-ir cells, “NPY-ir” was still present in glucagon- and somatostatin-ir cells (Figure 1D). Thus in neonates NPY appears to be present in beta cells but the “NPY-ir” in alpha and delta cells is likely to be PYY or PP.

**Figure 2:**
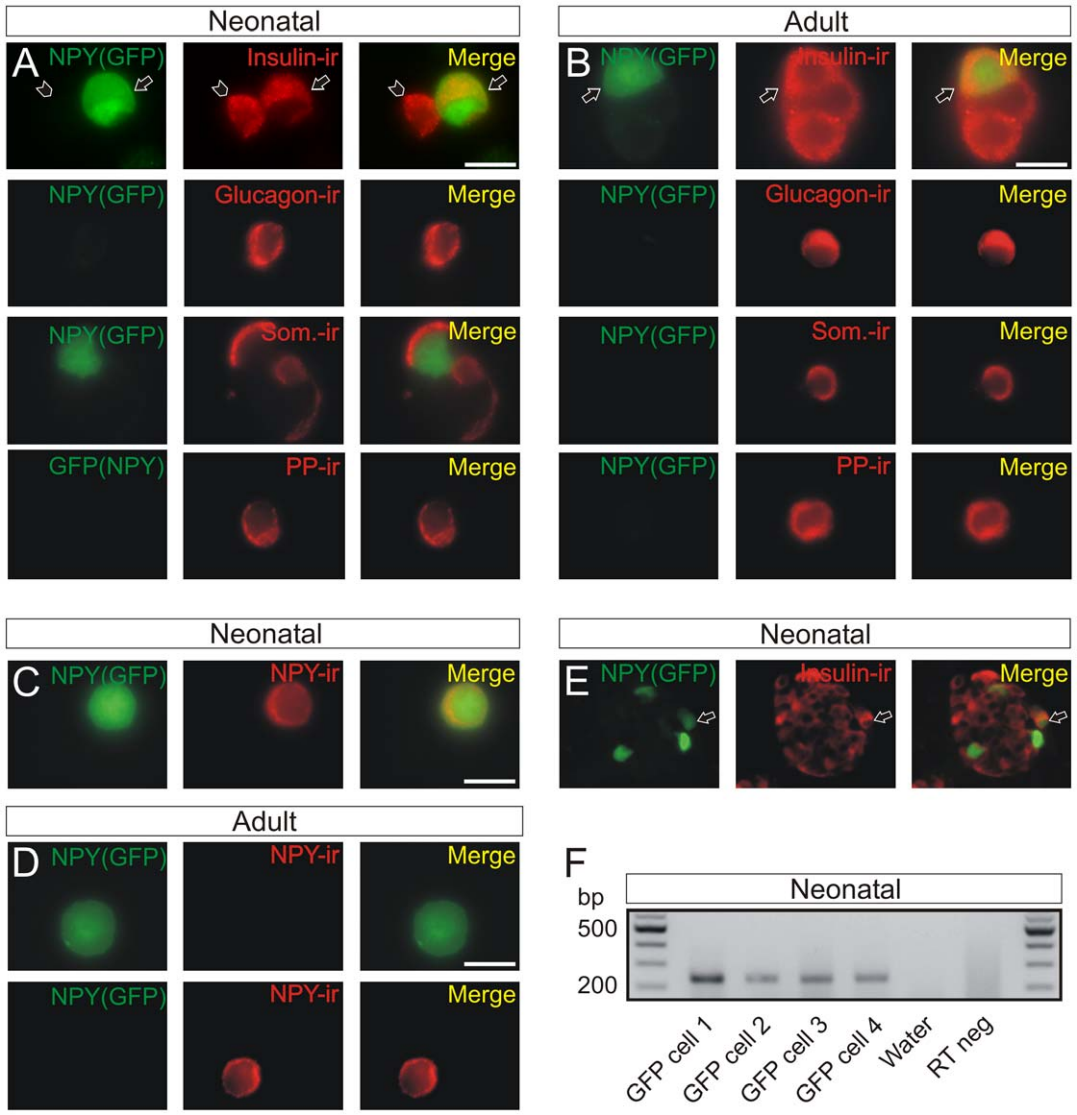
NPY is expressed in neonatal, but not adult, beta cells. (A). GFP expressing pancreatic cells from a neonatal (P0) NPY(GFP) transgenic mouse were stained for insulin, glucagon, somatostatin and pancreatic polypeptide. GFP was present only in cells that were insulin-ir (arrow; top panel). Also shown is a beta cell that does not express GFP (arrow head). (B). The same co-localization of GFP and insulin-ir (arrow; top panel) was seen in pancreatic cells from an adult (P25) NPY(GFP) transgenic animal. GFP was not expressed in glucagon-, somatostatin- or pancreatic polypeptide-ir cells. (C). GFP expressing cells from a neonatal (P0) NPY(GFP) animal are also NPY-ir. (D). Conversely none of the GFP cells from an adult (P39) NPY(GFP) animal were NPY-ir (top panel) even though NPY-ir cells were present in the culture (lower panel). (E). Thin section of a pancreatic islet from a P0 NPY(GFP) mouse showing that many GFP expressing cells are also insulin-ir (arrow indicates one example). Scale bars 10 µm (A–D); 50 µm (E). (F). Single cell RT-PCR showing that GFP expressing cells from a neonatal (P0) NPY(GFP) animal also contain insulin mRNA. Shown are 4 cells, and two controls (“Water” and “RT neg” which lacked either cellular material or reverse transcriptase, respectively). A single amplicon is present at the expected size for insulin (241 bp).

Given these complications two additional approaches were used to confirm the distribution of NPY expression. First, pancreatic cells were isolated from a BAC transgenic mouse in which GFP is expressed in cells that synthesize NPY [19]. In cultures from neonatal animals, GFP expression was found only in insulin-ir cells but was absent from glucagon-, somatostatin- and PP-ir cells (Figure 2A). A similar result was observed in islet cells from adult animals (Figure 2B). These results are in agreement with the data from the NPY knockout animals and indicate that in neonates NPY expression is limited to beta cells. However in adult islets a discrepancy exists; GFP expression is seen in insulin-ir cells but NPY-ir was absent from insulin-ir cells. To reconcile these observations I considered the possibility that it might be due to the fact that GFP is a cytoplasmic protein that does not enter secretory granules. If NPY synthesis were to cease during early post-natal development it is possible that cytoplasmic GFP would persist but that no NPY-ir would be observed.

To test this idea neonatal and adult islet cells from the BAC NPY(GFP) mice were stained for NPY. If a developmental switch occurred the prediction was that neonatal GFP cells would stain for NPY, but that adult GFP cells would not. This was indeed the case. As shown (Figure 2C) neonatal GFP cells were NPY-ir (n = 14 cells). However in adults none of the GFP-expressing cells were NPY-ir (n = 24 cells) although “NPY-ir” islet cells were present (Figure 2D). The latter are likely to be the putative PYY- or PP-expressing cells described above.

Thus after birth, the expression of NPY appears to be restricted to neonatal beta cells. As noted previously [4] a marked reduction in NPY expression occurred during the first postnatal week (only 12% (3/26) insulin-ir cells were NPY-ir at P5; not shown). Co-localization of GFP expression and insulin-ir was also seen in thin sections of pancreatic tissue from neonatal animals (Figure 2E) indicating that NPY-ir is expressed by beta cells *in situ* as well as *in vitro*.

Finally, if the neonatal NPY(GFP) expressing cells were indeed beta cells they should contain insulin mRNA. Single cell RT-PCR experiments confirmed that this was the case in all 8 cells examined (Figure 2F). Thus multiple lines of evidence are consistent with the idea that NPY is present in a population of beta cells early in post-natal development.

### Co-expression of insulin and neuropeptide Y in the INS-1 pancreatic beta cell line

**Figure 3:**
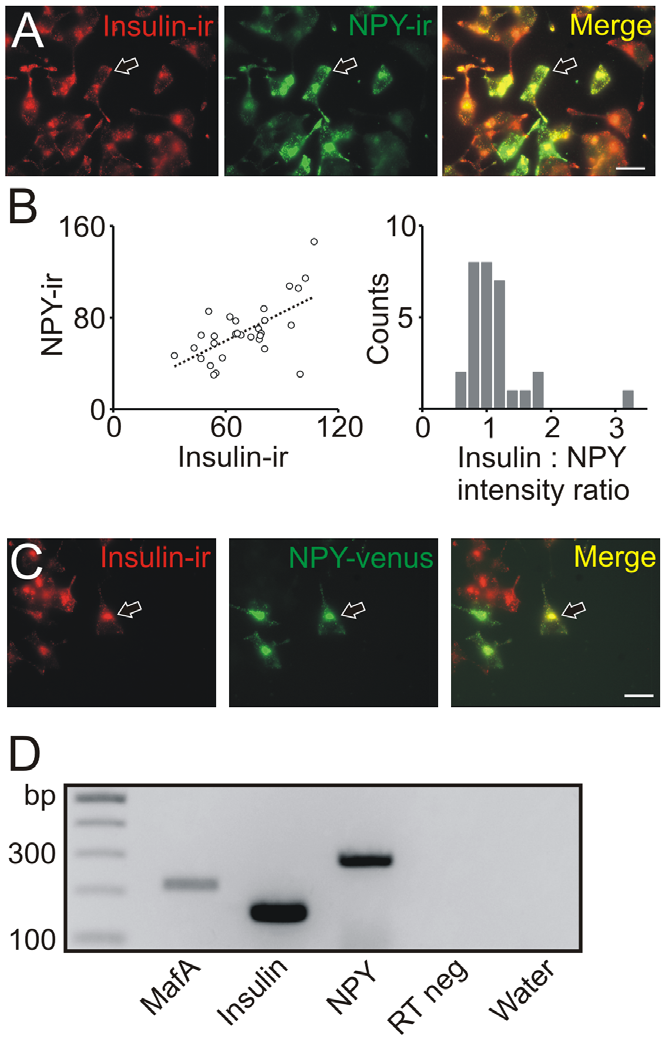
The INS-1 832/13 beta cell line synthesizes neuropeptide Y. (A). INS-1 cells were examined for both insulin- and NPY-ir. The merged image shows that the insulin- and NPY-ir co-localizes in individual cells (arrow). Scale bar 20 µm. (B). Quantification of the fluorescent signal from immunoreactive puncta. Left panel; a plot of fluorescence intensities indicates a positive correlation between the level of NPY- and insulin-ir consistent with the coexpression of NPY and insulin in individual granules (n = 30 granules from 6 cells, R = 0.62). Right panel; the distribution of the punctal signals. (C). INS-1 cells were transfected with a venus tagged NPY prohormone then stained for insulin-ir. Punctate NPY-venus fluorescence indicates that INS-1 cells have the ability to process the NPY prohormone. Scale bar 20 µm. (D). RT-PCR experiment showing the generation of amplicons consistent with the presence of MafA, insulin and NPY mRNA in INS-1 cells. All primers were intron spanning.

To examine the secretion of neuropeptide Y the INS-1 beta cell line was used. When INS-1 832/13 cells were stained with an NPY antibody, punctate NPY-ir was observed in the cell body and in the tips of the processes. All INS-1 cells were insulin-ir and the majority of the NPY-ir puncta were co-localized with the insulin-ir puncta (Figure 3A). Quantification of the fluorescent signal in the red and green channels indicated that individual puncta contained both NPY- and insulin-ir and that there was a positive correlation in the relative intensities of the two signals (Figure 2B, left panel) consistent with the co-expression of NPY and insulin in single granules. A histogram of the intensity ratios had a single peak (Figure 3B, right panel). These results are in agreement with the co-localization of NPY and insulin seen in primary beta cells (Figure 1B,C).

Next INS-1 cells were transfected with a plasmid encoding the NPY prohormone in which venus was fused to the carboxyl terminus of NPY. In transfected cells fluorescent puncta were seen in the cell body and at the tips of the processes (Figure 3C). Thus INS-1 cells contain the synthetic pathways needed for processing of the NPY prohormone.

Finally RT-PCR experiments confirmed that INS-1 832/13 cells contained both rat insulin and NPY mRNA. As expected they also expressed the MafA gene which encodes a transcription factor characteristic of beta cells (Figure 3D). These results are consistent with previous studies [20], [21] and the idea that INS-1 cells, like beta cells, synthesize NPY.

### Neuropeptide Y release monitored from INS-1 beta cells with the FMRFamide tagging technique

To determine whether NPY is secreted from beta cells in an activity-dependent manner the FMRFamide tagging technique was used [22]. In this approach the cells are transfected with several plasmids. One encodes the NPY prohormone which has been tagged with the coding sequence for the peptide FMRFamide. Expression of the tagged prohormone results in the synthesis of NPY and FMRFamide. Since these peptides are present on the same prohormone they are subsequently co-packaged into the same dense core granules. The cells are also transfected with a plasmid encoding the ionotropic FMRFamide receptor (and GFP to identify the transfected cells). When secretion is evoked by a voltage clamp depolarization the released FMRFamide activates the FMRFamide receptors on the same cell leading to an inward autaptic secretory current. The release of FMRFamide is thus used as a marker for the co-secretion of NPY. The advantage of this method is that it can follow peptide secretion on a rapid timescale and can detect release from single granules [22], [23].

**Figure 4:**
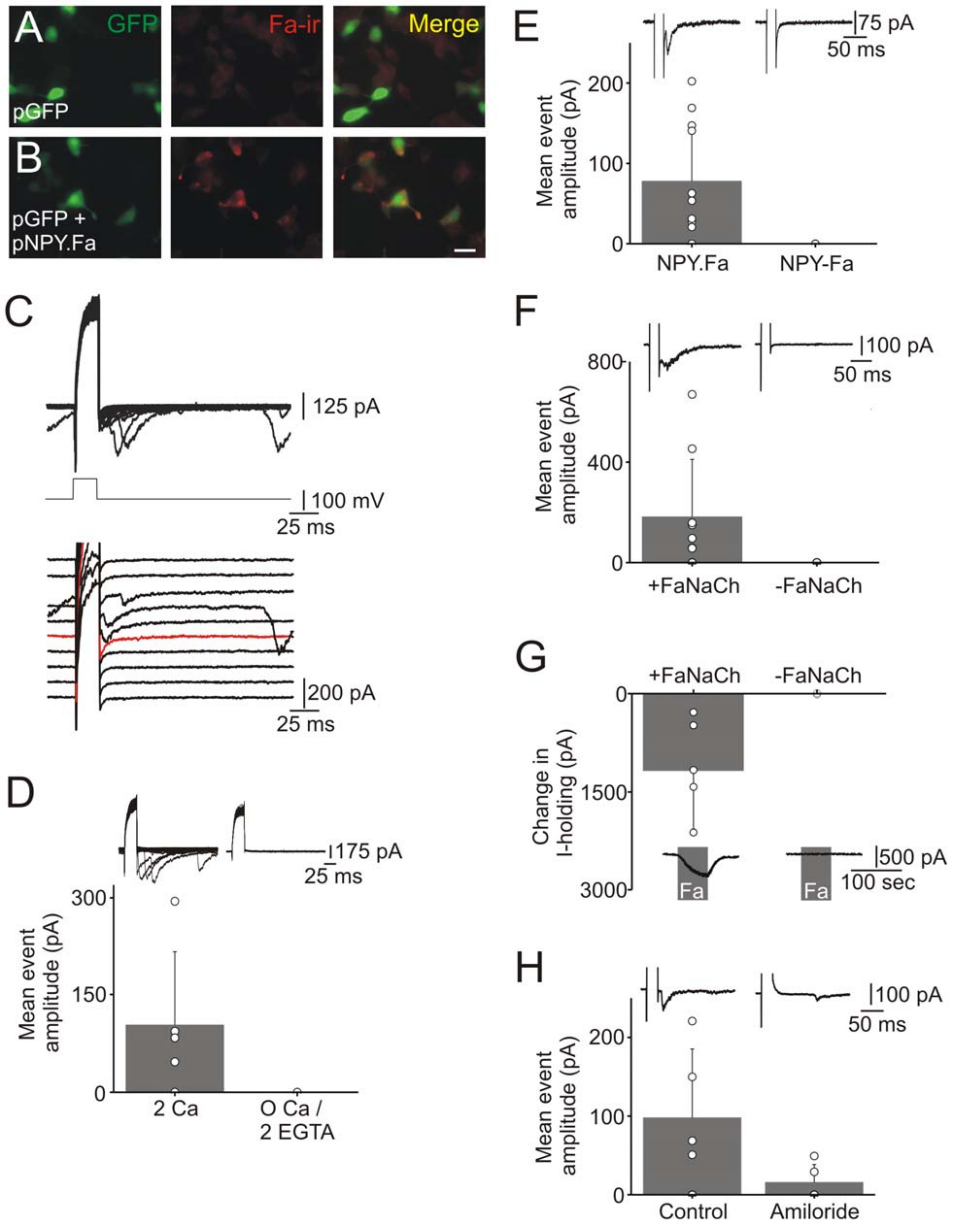
Neuropeptide Y release monitored from INS-1 beta cells with the FMRFamide tagging technique. (A). INS-1 cells that were transfected with GFP did not show any FMRFamide-ir. (B). INS-1 cells co-transfected with both GFP and the FMRFamide tagged prohormone were FMRFamide-ir. Scale bar 20 µm. (C). Example of secretory currents recorded from a cell that was transfected with the ionotropic FMRFamide receptor and the FMRFamide tagged NPY prohormone. Secretion was evoked with a train of 100 depolarizations at 5 Hz. Each depolarizing step lasted 20 ms and was from −80 to +20 mV. Sample traces (below; first 10 depolarizations) show that secretion was first detected following the fifth depolarization (red trace). (D). Secretory currents were not evoked from transfected cells when calcium was removed from the extracellular solution (and 2 mM EGTA was added). Secretion was evoked as in (C) except that the depolarization lasted 25 ms and was from −60 to +20 mV (n = 5 cells, mean ± SD). Sample recordings are shown above. (E). Secretory currents were consistently evoked from cells that were transfected with the FMRFamide receptor and a FMRFamide tagged NPY prohormone (“NPY.Fa”). In contrast no secretory events were observed in cells transfected with the FMRFamide receptor and a tagged prohormone in which the cleavage of FMRFamide was prevented (“NPY-Fa”). Cells were stimulated with the protocol described in (C). Sample recordings are shown above (n = 11–12 cells; mean ± SD). (F). Secretory events were evoked from cells co-transfected with NPY.Fa and the FMRFamide receptor (“+FaNaCh”) but were not evoked from cells transfected only with NPY.Fa (“−FaNaCh”). Cells were stimulated as in (C). Sample recordings are shown above (n = 6–9 cells, mean ± SD). (G). Application of exogenous 400 nM FMRFamide evoked an inward current only in cells that were transfected with both GFP and the ionotropic FMRFamide receptor (n = 4–5 cells; mean ± SD). Thus the secretory currents require the release of FMRFamide and INS-1 cells do not express an endogenous ionotropic FMRFamide receptor. (H). The mean amplitude of the secretory currents was lower in cells stimulated in the presence of 100 µM amiloride, a drug that inhibits the ionotropic FMRFamide receptor. Cells were stimulated with a train of 50 depolarizations using the protocol described in (D). Sample recordings are shown above (n = 5 cells, mean ± SD; n = 5 cells; P = 0.067, Mann-Whitney test).

To confirm that INS-1 cells can synthesize FMRFamide, cells were transfected with the FMRFamide-tagged NPY prohormone and GFP. FMRFamide-ir was observed in co-transfected cells but not in cells expressing only GFP (Figure 4A,B). Thus INS-1 cells do not contain an endogenous FMRFamide-like peptide.

When transfected cells were stimulated with a train of depolarizing steps, rapid inward currents were observed (Figure 4C). Removal of extracellular calcium prevented the appearance of the events (Figure 4D), consistent with the idea that they are due to calcium-dependent transmitter release. Four types of experiments indicated that these events were due to secretion of the FMRFamide tag. First, no events were detected from cells that were transfected with a tagged NPY prohormone in which FMRFamide was irreversibly fused to the C-terminus of NPY by removing the dibasic cleavage signal (Figure 4E). The absence of secretory events is expected because the ionotropic FMRFamide receptor is not activated by extended peptides like NPY-FMRFamide [24]. Second, the events required expression of the ionotropic FMRFamide receptor because they were not observed when cells were transfected only with the FMRFamide tagged NPY prohormone. However events were seen in sister cultures transfected with the tagged prohormone and the receptor (Figure 4F). Third, exogenous application of FMRFamide evoked an inward current only in cells that were transfected with the FMRFamide receptor, but not in untransfected cells (Figure 4G). Fourth, the amplitude of the secretory events was reduced in the presence of 100 µM amiloride (Figure 3H; −98±87 pA *vs.* 16±23 pA; mean ± SD; n = 5 cells; P = 0.067), a drug that inhibits the iontropic FMRFamide receptor [25]. These experiments indicate that generation of the secretory currents requires the release of FMRFamide, and that INS-1 cells do not endogenously express a FMRFamide-activated receptor.

### Release of a neuropeptide co-transmitter occurs with rapid kinetics from INS-1 beta cells

**Figure 5:**
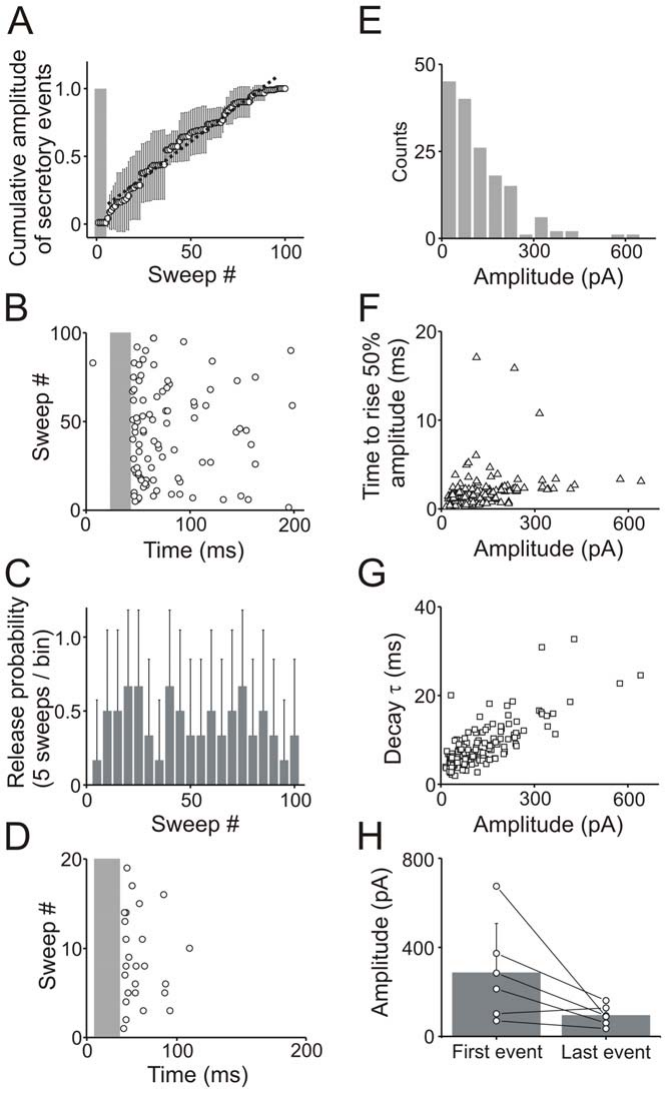
Release of a peptide co-transmitter from INS-1 beta cells occurs with rapid kinetics. (A). Plot of the cumulative amplitude of the secretory events *vs.* time from INS-1 cells that were stimulated with a train of 100 depolarizations (same protocol as Figure 4C). After a threshold is passed (grey bar; ∼5 depolarizations at this frequency), secretion occurs at a constant rate as indicated by the linear fit of the data (n = 6 cells, mean ± SD, R = 0.99). (B). Plot of the occurrence of individual secretory events. Although some events appear soon after the depolarizing step (grey bar) many events are not tightly locked to the depolarizing step (n = 6 cells). (C). Release probability did not vary substantially during a train of 100 depolarizations (stimulation protocol as in Figure 4C). Values are presented in 5 sweep bins (n = 6 cells; mean ± SD). (D). A lack of tight temporal coupling was also seen in the perforated patch configuration (n = 4 cells stimulated with 22 depolarizations from −80 to +20 mV at 1 Hz). (E–G). Plot of the amplitude, rise time and decay time constants of the secretory events recorded in the perforated patch configuration (n = 4 cells). (H). The amplitude of the first and last secretory events triggered by a train of 100 depolarizations at 5 Hz (n = 6 cells; mean ± SD; NS; P = 0.08, paired two-tailed t test).

When transfected INS-1 cells were stimulated with a train of 20 ms depolarizations at 5 Hz, secretory events began after a delay. A plot of the cumulative amplitude of the secretory events *vs.* time was linear indicating that once a threshold was reached, secretion occurred at a constant rate (Figure 5A). The secretory events were also not tightly coupled to the depolarizing step (Figure 5B) and the release probability did not vary substantially during a train of 100 depolarizations (Figure 5C). The lack of tight temporal coupling was seen both in whole cell (Figure 5B) and perforated patch configuration (Figure 5D). Thus it is unlikely to be due to dialysis of the cell interior. In fact these features are characteristic of peptide secretion and have been seen in a number of (neuro)endocrine cell types [26], [27].

The amplitude of the peptidergic secretory currents showed a wide variability (Figure 5E; 124±39 pA; mean ± SD, n = 4 cells). Most events had a rise time of a few milliseconds (Figure 5F, 1.4±0.5 ms; mean time to rise to half-amplitude ± SD, n = 4 cells). This value sets an upper estimate on the time course of the escape of the peptide tag from a fusing secretory granule. The secretory currents also decayed rapidly (Figure 5G, 8.7±1.9 ms, mean decay time constant ± SD, n = 4 cells). Although peptide release was most effectively triggered by a train of depolarizations, release did not facilitate. Thus when the amplitude of the first and last events in a train of 100 depolarizations were compared there was actually a trend towards a decrease although this was not statistically significant (Figure 5H).

**Figure 6:**
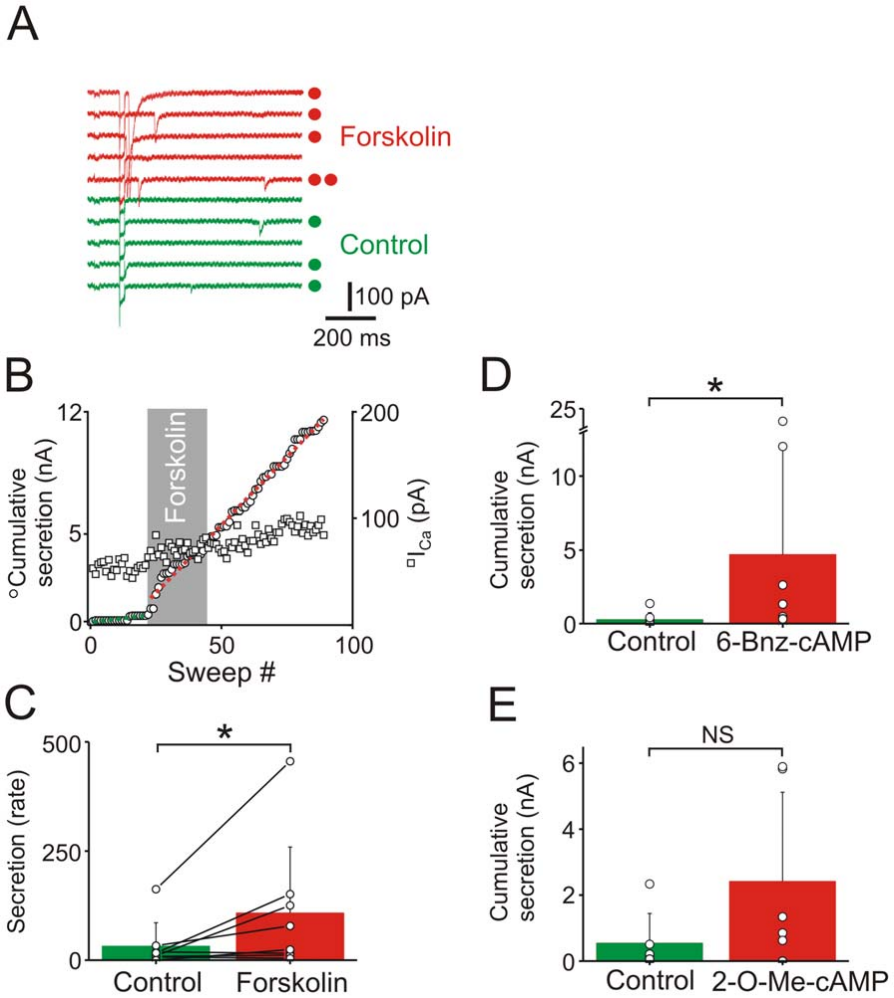
Release of a peptide co-transmitter from INS-1 beta cells is potentiated by cAMP. (A). Example of secretory events recorded from a transfected INS-1 cell before and after the application of 5 µM forskolin. The number of secretory events are indicated by the filled symbols to the right of the records. Shown are sections of 5 consecutive traces before (sweeps 1–5) and 5 consecutive traces after (sweeps 58–62) the application of forskolin. (B). Example experiment showing the time course of the potentiating effect of 5 µM forskolin on peptide secretion (open circles; same cell as in A). Square symbols show the amplitude of the depolarization-evoked calcium current over the course of the experiment. (C). Group data showing the effect of forskolin (n = 8 cells). The effect was quantified by comparing the gradient of the time-cumulative event amplitude relationship before and after the addition of 1–5 µM forskolin (n = 8 cells; mean ± SD; P<0.05, paired one-tailed t test). Cells in (A–C) were stimulated using the perforated patch technique at 0.1 or 1 Hz with a 20 ms depolarizing step from −80 to +20 mV. (D). Inclusion of 100 µM N^6^-benzoyl-cAMP, a selective activator of PKA, in the whole cell patch pipette solution significantly potentiated peptide secretion (n = 8 cells, mean ± SD; P<0.05, Mann-Whitney test). (E). Inclusion of 100 µM 2′-O-Me-cAMP, an EPAC activator, in the pipette solution did not significantly increase peptide secretion (n = 6 cells, mean ± SD; P<0.05, Mann-Whitney test). Cells in D and E were stimulated as described (Figure 4C).

Insulin release from beta cells is potentiated by an increase in the intracellular levels of cAMP [28], [29]. The FMRFamide tagging technique was then used to determine whether peptide release was similarly modulated. Application of 5 µM forskolin led to an increase in the number of peptidergic secretory events (Figure 6A). As seen in the example experiment shown in Figure 6B, forskolin resulted in a marked and sustained increase in peptide secretion monitored as the cumulative amplitude of the secretory events *vs.* time. The effect of forskolin was subsequently quantified by comparing the rate of secretion before and after forskolin addition. The group data revealed that forskolin led to a significant increase in peptide secretion (Figure 6C). In beta cells a rise in intracellular cAMP elevates insulin release *via* both PKA and EPAC-dependent pathways [30]. To test whether these pathways can regulate NPY/FMRFamide release, cAMP analogues that selectively activate either PKA or EPAC were included in the patch pipette solution. Depolarizing trains were applied 2 mins after break-in and evoked peptidergic events were quantified from interleaved control and experimental cells. Intracellular application of 100 µM N^6^-benzoyl-cAMP, a selective activator of PKA [31], was associated with a significant increase in peptide secretion (Figure 6D). Conversely 100 µM 2′-O-Me-cAMP, an EPAC activator [32], 2006), did not increase secretion, although a trend towards elevated release was observed (Figure 6E). These results suggest that activation of PKA is able to potentiate the release of both insulin and peptide co-transmitters from pancreatic beta cells.

## Discussion

Here I show that neonatal pancreatic beta cells synthesize neuropeptide Y as a co-transmitter in addition to the hormone insulin. This conclusion is based upon three observations. First, NPY-ir cells are also insulin-ir, the synthesis of the latter hormone being a characteristic marker of beta cells. No NPY-ir is seen in beta cells from NPY knockout mice confirming the immunoreactive signal is due to authentic NPY. Second, pancreatic GFP-expressing cells from a NPY(GFP) transgenic mouse are insulin-ir but do not stain with markers of alpha, delta or F cells. Third, using single cell RT-PCR, the GFP-expressing cells were shown to contain insulin mRNA. Although it has been reported that NPY is present in alpha- and somatostatin-containing cells, the present results are in agreement with other studies that localized NPY to beta cells [4], [12]. It should be noted that these conclusions apply to early post-natal development. By P25 there appears to a substantial down-regulation of NPY expression as noted by others [4]. In fact NPY expression appears to be characteristic of an early stage of beta cell development. For example the loss of the *neuroD* transcription factor in insulin-synthesizing cells results in immature beta cells and elevated islet NPY [13]. Nevertheless the postnatal loss of NPY does not seem to be irreversible because dexamethasone treatment can elevate islet NPY expression in adult rodents [33].

The idea that pancreatic endocrine cells can express co-transmitters in addition to their major polypeptide hormones is now widely accepted. Nevertheless the physiological role of these secreted co-transmitters is unclear and a subject of active investigation. However such co-transmitters are likely to be involved in modulating the secretion of pancreatic hormones by an autocrine or paracrine mode of action [34]. For example, GABA and ATP are synthesized by beta cells in addition the hormone insulin. Both molecules can regulate insulin secretion from beta cells [5], [6]. Likewise the classical transmitter glutamate is synthesized and secreted by alpha cells in addition to the hormone glucagon. Release of glutamate from these cells can increase glucagon release *via* autocrine feedback [35]. Alpha cells are also thought to express PYY [4]. Whether the other pancreatic endocrine cells, which include the somatostatin synthesizing delta cells, the pancreatic polypeptide synthesizing F cells and the ghrelin synthesizing epsilon cells also contain co-transmitters is less clear.

Using FMRFamide tagging the secretion of NPY was monitored from INS-1 beta cells. Previously the secretion of neuropeptides from the endocrine pancreas has been followed using methods which have low temporal resolution such as by RIA or the use of GFP-tagged fusion proteins. The non-specific NPY-ir that was seen in alpha, delta and F cells emphasizes the difficulties that can sometimes arise when peptide release is followed solely with techniques (such as RIA's) that rely on the use of antibodies. Using the FMRFamide tagging technique peptide secretion was found to occur *via* the regulated secretory pathway because it was calcium-dependent and required a depolarizing train. Previous studies using dexamethasone-treated RINm5F beta cells showed that NPY release was calcium-independent and not altered by potassium-induced depolarization [36]. Why peptide release differs between the two cell types is not clear. One possibility is that the constitutive release of peptide reflected a limitation in the number of dense core granules, with the “excess” peptide being diverted to the constitutive pathway [37].

Using FMRFamide tagging the majority of the peptidergic secretory events had a rise time of a few milliseconds which is comparable to the kinetics of peptidergic events observed in neuroendocrine chromaffin cells using the same approach [23]. This is curious because the primary transmitter(s) in chromaffin cells are the small molecules epinephrine and norepinephrine, while insulin plays this role in beta cells. The latter has a complex quaternary structure and is present in a crystalline form within the secretory granule. These features have consequences for hormone release. For example insulin is probably too large to exit through the initial fusion pore and even after full fusion the dissolution of the insulin core is thought to limit the release of the hormone [17]. In fact although the initial fusion pore is likely to delay the release of insulin, the escape of the small molecule co-transmitter ATP appears unhindered [18]. The rapid rise and decay times of the peptidergic secretory currents measured in this study imply that the escape of the FMRFamide peptide tag is not delayed. Thus small peptides like FMRFamide and perhaps NPY (molecular weight 599 and 4272, respectively) appear more similar in their release kinetics to classical transmitters than to hormones like insulin (molecular weight 5808). While it is possible that FMRFamide and NPY are present in granules that lack insulin, in both primary beta cells and in the INS-1 cell line, many insulin- and NPY-ir puncta were co-localized. Thus it seems likely that these transmitters are co-packaged in at least a subset of the dense core granules.

Peptide release from transfected INS-1 cells was elevated by forskolin and N^6^-benzoyl-cAMP, an activator of PKA. Previous work has shown that forskolin also increases the release of endogenous NPY from INS-1 cells [20]. While insulin secretion from beta cells is potentiated by cAMP *via* PKA-dependent and independent (EPAC) pathways, an EPAC activator did not significantly alter NPY/FMRFamide release. Whether this reflects a difference in the mechanisms that regulate peptide and insulin release is not clear. Overall there appear to be many similarities.

Although the role of the released NPY was not investigated in the present study, previous work has shown that exogenous NPY can inhibit insulin release [9] *via* the activation of NPY1 receptors [38]. If Y1 receptors are present on beta cells [39] this might represent a short feedback loop that could act to suppress an excessive or pathological release of insulin. One complication is that NPY-ir was not detectable in the adult pancreas. Thus under normal circumstances the endogenous agonist for these receptors is probably PYY. However a post-natal role for NPY could exist under pathological conditions. For example when glucocorticoid levels are elevated or during type II diabetes, the pancreatic levels of NPY are reported to increase [33], [40], [41].

If under physiological conditions NPY is mainly expressed in immature islets it is unlikely to play a significant role in regulating blood glucose levels since maturation of the pancreatic response to glucose is thought to take place after birth [42], [43]. What might be the function of pancreatic NPY if it is not usually present in the post-natal mouse? Since the global knockout of NPY is associated with an increase in islet size [10], in future work it might be informative to examine whether NPY is involved in regulating early pancreatic development.

## Materials and Methods

### Ethics statement

All procedures involving animal tissue were approved by the Institutional Animal Care and Use Committee of LSU Health Sciences Center, New Orleans (#2711).

### Cell preparations

INS-1 832/13 cells (kindly provided by Dr.'s Christopher Newgard (Duke University), Douglas Cavener (PSU) and Kim Pedersen (LSUHSC)) were maintained in RPMI/10% FCS containing 50 µM 2-mercaptoethanol, 1 mM sodium pyruvate and 1 mM HEPES. Cells were plated on poly-d-lysine coated coverslips (immunocytochemistry) or on uncoated 35 mm culture dishes (electrophysiology). Islet cells (P0–39) were isolated either from wild type C57BL/6J mice, a BAC transgenic line (Npy-hrGFP) in which cytoplasmic GFP is expressed in cells that synthesize NPY [19] or NPY knockout animals [44]. All lines were obtained from The Jackson Laboratory. Briefly, the pancreas was removed, cut into small pieces and incubated for 20–30 minutes in collagenase type XI at 37°C. The tissue was washed, re-suspended in 1 ml Histopaque-1077 and triturated. Culture medium (0.2 ml) was overlaid and the sample was centrifuged at 900 g for 12 minutes. Tissue in the supernatant (or handpicked islets in the case of adult animals) was collected and incubated in HEPES-buffered salt solution containing 1 mM EDTA for 5 minutes and then gently triturated to release single cells. The dispersed cells were plated on in the same culture medium as INS-1 cells.

### Electrophysiology

Whole cell and perforated patch clamp recordings were made with a Multiclamp 700A amplifier (Molecular Devices) as described [22], [23]. Cells were superfused with an extracellular solution containing (in mM) 135 NaCl, 3 KCl, 2 CaCl_2_, 1 MgCl_2_, 10 HEPES, and 5 glucose, pH 7.3, with NaOH. The pipette solution contained (in mM) 120 K-acetate, 15 KCl, 5 NaCl, 10 HEPES, 4 MgATP and 0.3 NaGTP, pH 7.2 with KOH. In experiments in which the calcium current was monitored the pipette solution contained (in mM) 120 Cs-acetate, 15 CsCl, 5 NaCl, 10 HEPES, 4 MgATP, 0.3 NaGTP, pH 7.2 with CsOH. Some recordings (Figure 5D–G; 6A–C) were made using the perforated patch technique in which the pipette solution contained 0.1 mg/ml amphotericin B (and lacked ATP and GTP). All experiments except Figure 4G were conducted at ∼33°C. Most chemicals were obtained from Sigma-Aldrich except for N^6^-benzoyl-cAMP and 2′-O-Me-cAMP (Biolog).

Pipette resistance was generally ∼6 MΩ and no compensation was applied. Access resistance was typically 15–25 MΩ. Secretory currents were analyzed using pClamp9 (Molecular Devices), OriginPro7 (Microcal Software, Northampton, MA) and Excel (Microsoft, Seattle, WA). Statistical significance was assessed using the Students t test or Mann-Whitney test as indicated.

FMRFamide tagging was as previously described [22]. Cells were transfected with 0.2 µg pGFP/0.65 µg pFaNaCh/0.65 µg pNPY.Fa using Lipofectamine 2000 (Invitrogen) and were used for experiments 48–72 hr following transfection. In Figure 4G, the FMRFamide receptor was expressed using a GFP-containing IRES vector.

### *In vitro* immunocytochemistry

Cells were fixed and stained as described [45]. Primary antibodies used were rabbit anti-NPY (1∶1000; T-4070; Peninsula Laboratories), rabbit anti-FMRFamide (1∶200; IHC8755; Peninsula Laboratories), guinea pig anti-insulin (1∶5000; I8510; Sigma), mouse anti-glucagon (1∶2000; G2654; Sigma), rat anti-somatostatin (1∶500; MAB354; Millipore), and rabbit anti-pancreatic polypeptide (1∶1000; AB939; Millipore). Secondary antibodies were donkey anti-rabbit Alexa 488 (1∶200; Invitrogen), donkey anti-guinea pig; donkey anti-mouse; donkey anti-rat and donkey anti-rabbit (conjugated to dylight 549; all used at 1∶100; Jackson ImmunoResearch). For double-staining experiments, antibodies were applied sequentially. Control experiments showed there was no significant bleed-through of the fluorescent labels or cross-reactivity between antibodies. In some experiments the cells were transfected with 0.65 µg pNPY-Venus [46]. Most images were obtained with a Nikon TE2000U microscope with a 60× (1.4 numerical aperture) oil-immersion objective and a Retiga 1300 monochrome camera and Image-Pro software. Data in Figure 1B,C were obtained using a Leica DM IRE2 confocal microscope with a 63× water immersion objective.

### *In situ* immunohistochemistry

Pancreatic tissue was fixed in 4% paraformaldehyde in PBS overnight at room temperature (RT) and stored at 4°C in PBS containing 30% sucrose. Tissue was embedded in cryomatrix medium and 20 µm frozen sections were prepared. Sections were fixed for 20 min in 4% paraformaldehyde, permeabilized (0.3% TritonX-100 in PBS for 15 min), maintained in blocking solution (PBS containing 0.05% Triton X-100 and 0.25% BSA) for 30 mins, then incubated overnight at 4°C in primary antibody in blocking solution. Slices were washed (six times for 10 min with PBS) and incubated with a secondary antibody for 90 mins at RT. After washing, the sections were mounted in Vectashield.

### Reverse transcription-PCR

RNA was isolated from rat INS-1 cells growing on a single 35 mm dish and RT-PCR was performed as described [45]. The forward NPY primer was 5′- GCTAGGTAACAAACGAATGGGG-3′, and the reverse NPY primer was 5′-CACATGGAAGGGTCTTCAAGC-3′ (288 bp product). The forward insulin primer was 5′-TGTGGTTCTCACTTGGTGGA-3′, the reverse insulin primer was 5′-CAGTGCCAAGGTCTGAAGGT-3′ (156 bp product). The forward MafA primer was 5′-TTCAGCAAGGAGGAGGTCAT-3′, the reverse MafA primer was 5′-CCGCCAACTTCTCGTATTTC-3′ (217 bp product). All primers were intron spanning.

### Single cell RT-PCR

Pancreatic cultures were prepared from P1 NPY-GFP mice and single GFP positive cells were aspirated into 5–10 µm diameter patch pipettes containing 1 µl DEPC-treated water. The subsequent steps largely followed those of van den Pol et al [19]. Briefly, the pipette contents were ejected into a PCR tube containing 2 µl 5× RT buffer, 0.5 µl RNasin (40 U/µl) and 4.5 µl water. Reverse primer was added (1 µl of 20 µM stock) and the mixture heated to 70°C for 5 mins then stored on ice. Reverse transcription reaction was performed by incubation at 42°C for 60 mins after the addition of 1 µl of 200 U/µl Moloney murine leukemia virus reverse transcriptase and 1 µl of 10 mM dNTPs. The reaction was terminated by incubation at 95°C for 5 min. RNA in an RNA : DNA duplex was digested by the addition of 0.5 µl of 2 U/µl RNase H and incubation at 37°C for 20 mins. Hot start PCR used an intron spanning primer for the mouse insulin2 prohormone (forward primer 5′-TTTGTCAAGCAGCACCTTTG-3′; reverse primer 5′-GCTGGTAGAGGGAGCAGATG-3′; 241 bp product). Each reaction contained 5 µl of 10× buffer, 2.5 µl of 20 µM primer stock, 1 µl of 5 U/µl Taq polymerase, 5 µl of the reverse transcription reaction, and water to a final volume of 50 µl. The PCR protocol was 94°C for 3 mins, then 94°C for 45 s, 55°C for 45 s, and 72°C for 60 s, for 35 cycles, and finally 72°C for 10 mins. Samples (20 µl) were analyzed on 2% agarose gels, photographed and the image was inverted for clarity. Controls included omission of reverse transcriptase (to reveal any genomic contamination) and aspirated bath solution (to test for non-specific amplification). Both controls were negative.
